# Magnesium Promotes Growth–Metabolism Balance in Juvenile Largemouth Bass (*Micropterus salmoides*) and Modulates Antioxidant–Inflammatory–Apoptotic Responses Under Heat Stress

**DOI:** 10.3390/antiox14121394

**Published:** 2025-11-23

**Authors:** Junjie Qin, Dongyu Huang, Hualiang Liang, Xiaoru Chen, Jiaze Gu, Mingchun Ren, Lu Zhang

**Affiliations:** 1 Wuxi Fisheries College, Nanjing Agricultural University, Wuxi 214081, China; 2023813049@stu.njau.edu.cn (J.Q.); lianghualiang@ffrc.cn (H.L.); 2024213006@stu.njau.edu.cn (J.G.); 2 Key Laboratory of Integrated Rice-Fish Farming Ecology, Ministry of Agriculture and Rural Affairs, Freshwater Fisheries Research Center, Chinese Academy of Fishery Sciences, Wuxi 214081, China; huangdongyu@ffrc.cn; 3 Tongwei Agricultural Development Co., Ltd., Key Laboratory of Nutrition and Healthy Culture of Aquatic, Livestock and Poultry, Ministry of Agriculture and Rural Affairs, Healthy Aquaculture Key Laboratory of Sichuan Province, Chengdu 610093, China; chenxr@tongwei.com

**Keywords:** magnesium, largemouth bass, growth performance, nutrient metabolism, heat stress

## Abstract

This study addressed the optimal magnesium (Mg) requirement for juvenile largemouth bass (*Micropterus salmoides*) and assessed the effects of dietary Mg supplementation on growth performance, nutrient metabolism, and alleviation of heat stress in it. In this study, six diets with varying Mg levels (1.01, 1.26, 1.78, 2.24, 2.35, and 2.51 g/kg), designated as MG1, MG2, MG3, MG4, MG5, and MG6, respectively, were formulated using MgSO_4_·7H_2_O as the Mg source. These diets were fed to juvenile *M. salmoides* (initial body weight 2.27 ± 0.02 g) for 8 weeks. The growth performance of the MG4 group was significantly improved. In addition, Plasma GLU, LDL-C, and TG levels were significantly reduced in the MG4 group, while plasma HDL-C levels were increased. In terms of gene expression, *glut2*, *g6pdh*, *ppar-γ*, *fas*, *elovl2*, *acc*, and *igf-1* were significantly upregulated in the MG4 and MG5 groups, while *g6pase* and *ppar-α* were significantly downregulated in the MG5 group. In the heat stress test, MG4 group exhibited enhanced antioxidant capacity, as evidenced by decreased plasma MDA levels and increased CAT activity, coupled with enhanced gill Na^+^/K^+^-ATPase activity. Gene expression results also showed that *il-10* and *bcl-2* were significantly upregulated in the MG4 group, while *nf-κb*, *ifn-γ*, *il-8*, *tnf-α*, *casp3*, *casp8*, *bax*, *jnk2* and *ask1* were significantly downregulated. Furthermore, the results of TUNEL immunofluorescence labeling analysis showed that the apoptotic index was significantly decreased in the MG2-MG6 groups. Overall, appropriate dietary Mg levels promoted growth performance, improved glucose metabolism, and induced lipid deposition in juvenile *M. salmoides.* Notably, Mg reduced oxidative damage by enhancing antioxidant enzyme activity, thereby modulating heat stress-induced Antioxidant–Inflammatory–Apoptotic of juvenile *M. salmoides.* Based on quadratic regression analysis of SGR and FCR, the optimal Mg requirement for juvenile *M. salmoides* was 2.04, and 2.15 g/kg, respectively.

## 1. Introduction

In the aquaculture industry, feed costs constitute over 70% of variable production costs [1], making the formulation of nutritionally balanced feeds critically important. Although minerals constitute only a small proportion of feed, they are indispensable for fish growth. Magnesium (Mg), as an essential cofactor in over 300 enzymatic reactions, exhibits species-specific requirements influenced by ontogenetic stage and environmental parameters [2]. For example, the optimum Mg requirement for juvenile hybrid sturgeon (*Acipenser schrenckii* ♀ × *Acipenser baerii* ♂) is 350–700 mg/kg, which improves antioxidant properties [3]. The best demand for magnesium in gibel carp (*Carassius auratus gibelio*) is 750 mg/kg, with concentrations exceeding 2900 mg/kg inducing adverse effects [4]. At the same time, the Mg content in freshwater and seawater varies considerably. Unlike marine fish that efficiently regulate Mg via seawater ingestion, freshwater species require exogenous Mg supplementation to compensate for environmental deficiency [5,6]. Considering the important role of Mg as a mineral, it is imperative to clarify the specific Mg requirements for various fish species.

The research of fish nutrient metabolism has progressed from optimal requirements of macronutrients to contemporary explorations of micronutrient synergism, metabolic pathway regulation, and multi-omics integrated application [7]. Minerals play a crucial role in the metabolism of nutrition in fish. For example, copper, zinc, and iron influence the balance between protein synthesis and degradation within cells by regulating metal transporter proteins [8]. The effect of Mg on nutrient metabolism is also critical. It is associated with protein synthesis and can indirectly influence total plasma protein (TP) levels [9]. Mg is also involved in insulin secretion and signaling pathways [10], which enhances glycolysis and glycogen synthesis while inhibiting gluconeogenesis in the blunt snout bream (*Megalobrama amblycephala*) [2]. However, Mg seems to have different effects on lipid metabolism in different fish. It was found that Mg increased lipid deposition in *Heteropneustes fossilis* [11], while the opposite was true for yellow catfish (*Pelteobagrus fulvidraco*) [12].

Global warming has led to rising temperatures and extreme heatwaves, posing a significant threat to fish physiology, behavioral patterns, and population dynamics [13]. As poikilotherms, fish are highly sensitive to temperature changes, which directly affect their survival and growth [14,15,16]. Therefore, in recent years, researchers have increasingly focused on the mechanisms of heat stress response in fish [17]. The role of Mg in alleviating heat stress in fish is multifaceted and involves various physiological and biochemical mechanisms. Elevated water temperatures lead to lower dissolved oxygen in the water, which promotes the production of reactive oxygen species (ROS), leading to oxidative stress [18,19]. As a cofactor for multiple antioxidant enzymes, Mg directly enhances ROS scavenging capacity while reducing oxidative damage and maintaining cellular homeostasis by stabilizing mitochondrial membrane structure, inhibiting calcium overload, and cross-linking cell membrane phospholipids. At the same time, oxidative stress weakens the immune function of fish and reduces their resistance to disease [20,21]. Dietary supplementation with Mg has been reported to reduce oxidative stress [22] and enhance immunity [23]. In addition, temperature changes interfere with the normal function of the sodium–potassium pump, affecting ion transport and disrupting the ionic balance inside and outside the cell [24,25]. Mg has a non-negligible role in maintaining ionic homeostasis and regulating osmotic pressure [26], which has been confirmed in the study of freshwater shrimp (*Macrobrachium olfersii*) [27]. ROS production and abnormal function of the Na^+^/K^+^-ATPase in turn trigger apoptosis [28,29]. Mg deficiency exacerbates apoptosis and impairs intestinal structural integrity in *C. idella* [30]. In summary, Mg acts as a key nutrient for fish to combat heat stress through its synergistic effects of “antioxidant–inflammatory–apoptotic,” playing a central role particularly in maintaining redox balance. An in-depth study of the role of minerals in alleviating heat stress in fish is essential for ensuring the survival and health under high temperature.

As a globally important freshwater economic fish species, *M. salmoides* has become a model species for aquatic nutrition and adversity physiology research due to its high protein requirement, rapid growth characteristics, and temperature sensitivity [31,32]. However, three critical knowledge gaps currently hinder the optimization of its Mg nutrition: unknown Mg requirement, nutrient metabolism interactions, and Mg-mediated mechanisms under heat stress. To address these gaps, this study directly investigates the effects of graded dietary Mg levels on juvenile *M. salmoides*, with three specific objectives: (1) determine the optimal dietary Mg requirement; (2) clarify Mg’s role in regulating nutrient metabolism (protein, lipid, and glucose homeostasis); (3) elucidate the mechanisms by which Mg alleviates heat stress. By filling these critical knowledge gaps, our findings will provide the first species-specific Mg requirement guidelines for *M. salmoides* feed formulation, enhancing both production efficiency and stress resilience in this economically vital species.

## 2. Materials and Methods

### 2.1. Experiment Diet

The basal diet formulation used in the experiment is presented in Table 1. Six experimental diets with Mg content of 1.01, 1.26, 1.78, 2.24, 2.35, and 2.51 g/kg were formulated by adding MgSO_4_·7H_2_O to the basal diet in a gradient. These diets were designated as MG1 (control), MG2, MG3, MG4, MG5, and MG6. The MgSO_4_·7H_2_O was purchased from Shanghai Macklin Biochemical Technology Co., Ltd. (Shanghai, China). During diet preparation, all ingredients were ground and sieved (80-mesh). The powdered ingredients were then homogenized with water and oil according to the formulation. The resulting mixtures were pelletized using an F-26(II), South China University of Technology (Guangzhou, China), and then air-drying. The feed proximate composition analysis was performed as follows: Crude protein was measured by a Hanon K1100 automatic instrument (Jinan, China). Crude lipid was measured by a Hanon SOX606 auto fat analyzer (Jinan, China). Gross energy was measured by an IKA C6000 oxygen bomb calorimeter (Guangzhou, China).

### 2.2. Fish Culture

In this study, 360 juvenile *M. salmoides* were procured from Zhengda Aquatic Products Co., Ltd (Huzhou, China). Culture experiments were conducted within an indoor recirculating aquaculture system (equipped with temperature regulation and purification capabilities, each tank with a capacity of about 270 L) provided by ZHONGKEHAI Recycling Water Aquaculture System Co., Ltd. (Qingdao, China). The experimental site was the Feed Observation Station of the Freshwater Fisheries Research Center, Chinese Academy of Fishery Sciences (CAFS) (Yixing, China). Following a two-week acclimation period with the control diet, all fish underwent 24 h fasting before the formal experiment. Healthy juvenile *M. salmoides* (initial body weight of 2.27 ± 0.02 g) were randomly allocated into six experimental groups (three replicates per group, 20 fish per replicate). Throughout the 8-week culture period, the feed was administered thrice daily (07:30, 12:30, and 17:30) to visual satiety. Daily monitoring included feeding assessment and mortality recording. Metabolic wastes were removed using a plastic vacuum cleaner. Water quality parameters were controlled at: temperature 28 ± 2 °C, pH 7.4 ± 0.4, DO ≥ 6 mg/L, NH_3_-N < 0.05 mg/L. And the light/dark ratio was 1:1.

### 2.3. Sample Collection

Following the culture period, all fish underwent 24 h fasting prior to sampling. Three fish per tank were anesthetized (MS-222) for blood collection, with immediate centrifugation (4000 rpm, 10 min, 4 °C) to obtain upper plasma for biochemical testing. After weighing and measuring the body length, their liver was collected and placed in freezing tubes and stored at −80 °C for the determination of the mRNA expression levels related to the nutrient metabolism gene. Furthermore, three fish were taken from each tank and frozen at −20 °C to perform whole-body composition analysis.

### 2.4. Heat Stress Trial

A total of 180 fish, one group of 30 fish, were divided into six groups fed six different Mg concentration diets for the heat stress trial. The water temperature was increased from 28 °C to 33 °C (0.5 °C/h) and then maintained continuously at 33 °C for seven days. Three fish per tank after heat stress were anesthetized for blood collection, with immediate centrifugation (4000 rpm, 10 min, 4 °C) to obtain upper plasma for the determination of ionic concentration and antioxidant indices. Three more fish were taken for their gill tissues, and a portion of the gill tissue samples was placed in freezing tubes for the determination of Na^+^/K^+^-ATPase activity, immunity, and apoptosis-related gene mRNA expression levels. The other part of the gill tissue samples was stored in 4% paraformaldehyde for TUNEL immunofluorescence labeling analysis.

### 2.5. Laboratory Analysis

Crude protein and lipid content of fish were determined in the same way as for feeds. Details of moisture, ash, and other chemical analysis (plasma biochemical indices, plasma antioxidant indices, plasma ion concentrations, and gill Na^+^/K^+^-ATPase activity) are shown in Table 2.

Total RNA from liver and gill tissues was extracted by the FreeZol Reagent kit (Vazyme Biotech Co., Ltd., Nanjing, China) and the RNA quality was adjusted to 60 ng/μL using the NanoDrop 2000 spectrophotometer (Shanghai, China). Subsequently, RT-PCR was performed, as detailed in Table 2. Relative mRNA expression was determined according to Pfaffl’s mathematical model. *gapdh* was used as an internal reference gene. Primers were all synthesized by Shengong Bioengineering Co., Ltd. (Shanghai, China). Primer details are summarized in Table 3. The 4% paraformaldehyde-fixed gill tissues were submitted to Wuhan Servicebio Technology Co., Ltd. (Wuhan, China) to be analyzed for apoptosis by TUNEL immunofluorescence labeling.

### 2.6. Statistical Analysis

Prior to statistical analysis, variance homogeneity was validated through Levene’s test. IBM SPSS Statistics 20 software was used for performing ANOVA. Graphical representations were generated using OriginPro 2024 software. Microsoft Excel is used to perform the quadratic regression analysis. Data were shown as mean ± SD, with Tukey’s test defining significance at *p* < 0.05.

## 3. Results

### 3.1. Growth Performance

**Figure 1:**
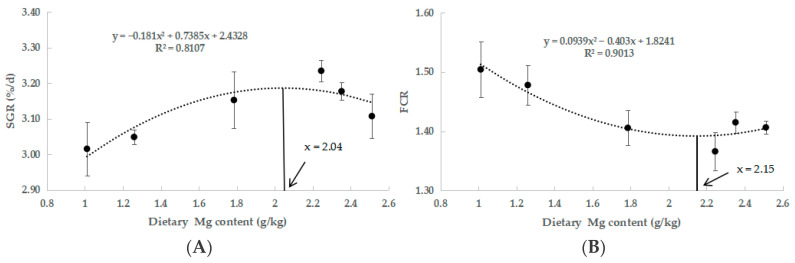
Quadratic regression analysis of FCR and SGR in response to varying Mg levels. (**A**) SGR, (**B**) FCR.

Table 4 presents the results of growth performance. The MG4 group exhibited significantly higher final body weight (FBW), specific growth rate (SGR), and weight gain rate (WGR) (*p* < 0.05). Additionally, the feed conversion ratio (FCR) was significantly lower in the MG4 group (*p* < 0.05). Through quadratic regression analyses of SGR and FCR, we established the optimal Mg requirement for juvenile *M. salmoides* at 2.04 and 2.15 g/kg (Figure 1).

### 3.2. Whole-Body Composition

Table 5 presents the whole-body composition analysis results. Crude lipid content showed an increasing trend with elevated dietary Mg supplementation, with the MG6 group demonstrating significantly higher values (*p* < 0.05). Nevertheless, no significant variations were found in moisture, crude protein, and ash content (*p* > 0.05).

### 3.3. Plasma Biochemical Indices

Table 6 presents the results of plasma biochemical indices. GLU levels tended to decrease and reached a significant minimum in the MG4 group (*p* < 0.05). LDL-C and TG levels were significantly reduced in the MG2–MG6 groups, while HDL-C was markedly elevated (*p* < 0.05). TC and TP levels showed no significant differences (*p* > 0.05).

### 3.4. Genes Related to Glucose Metabolism in the Liver

**Figure 2:**
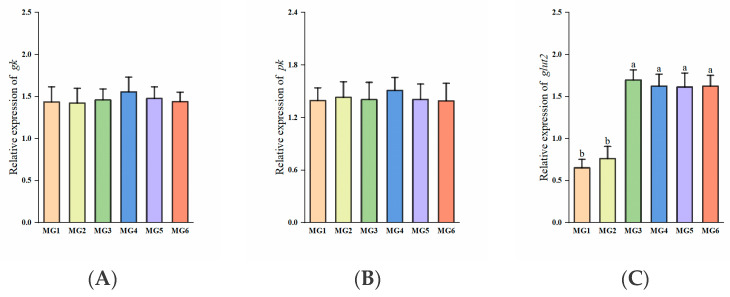

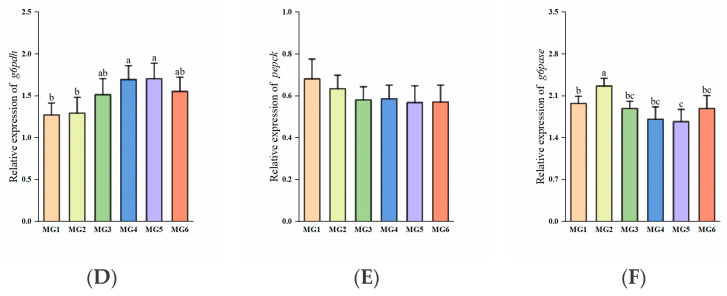
Expression of glucose metabolism-related genes in the liver (n = 9): (**A**) *gk*, (**B**) *pk*, (**C**) *glut2*, (**D**) *g6pdh*, (**E**) *pepck*, (**F**) *g6pase*. Statistically significant variations (*p* < 0.05) were indicated by distinct lowercase alphabetical superscripts, with unmarked values demonstrating no significant difference.

Significantly higher *glut2* expressions were observed in the MG3-MG6 groups (*p* < 0.05, Figure 2C). The expressions of *g6pdh* were significantly higher in the MG4 and MG5 groups (*p* < 0.05, Figure 2D). The expression of *g6pase* was lowest in the MG5 group (*p* < 0.05, Figure 2F). No significant differences were observed in the expressions of *gk*, *pk*, and *pepck* (*p* > 0.05, Figure 2A,B,E).

### 3.5. Genes Related to Lipid Metabolism in the Liver

**Figure 3:**
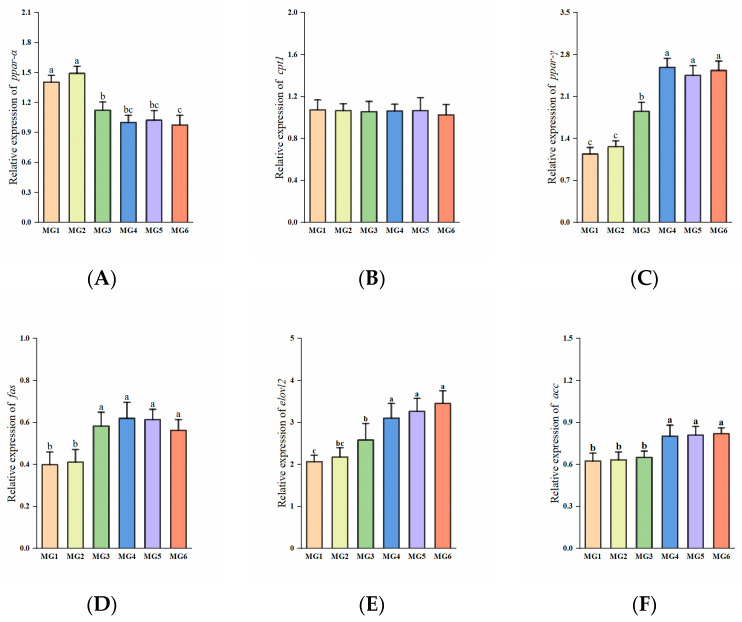
Expression of lipid metabolism-related genes in the liver (n = 9): (**A**) ppar-α, (**B**) cpt1, (**C**) ppar-γ, (**D**) fas, (**E**) elovl2, (**F**) acc. Statistically significant variations (*p* < 0.05) were indicated by distinct lowercase alphabetical superscripts, with unmarked values demonstrating no significant difference.

The expressions of *ppar-α* in the MG3-MG6 groups were significantly reduced (*p* < 0.05, Figure 3A). No significant intergroup differences were observed in *cpt1* expressions (*p* > 0.05, Figure 3B). The MG3–MG6 groups exhibited significant upregulation of *ppar-γ*, *fas*, and *elovl2* expressions (*p* < 0.05, Figure 3C–E). Furthermore, the expressions of *acc* in the MG4–MG6 groups were markedly elevated (*p* < 0.05, Figure 3F).

### 3.6. Genes Related to Protein Metabolism in the Liver

**Figure 4:**
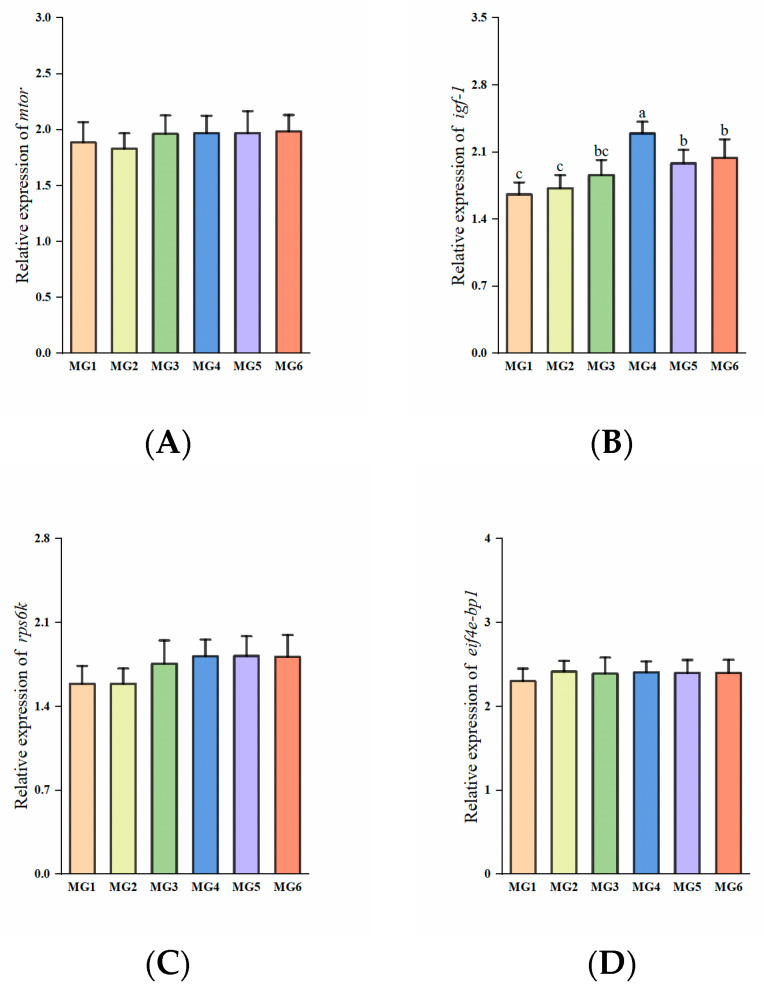
Expression of protein metabolism-related genes in the liver (n = 9): (**A**) *mtor*, (**B**) *igf-1*, (**C**) *rps6k*, (**D**) *eif4e-bp1*. Statistically significant variations (*p* < 0.05) were indicated by distinct lowercase alphabetical superscripts, with unmarked values demonstrating no significant difference.

The expression of *igf-1* was the highest in the MG4 group with an initial upregulation followed by progressive downregulation (*p* < 0.05, Figure 4B). Both the expressions of *mtor* and *rps6k* were elevated in the MG3-MG6 groups (*p* > 0.05, Figure 4A,C). No significant differences observed in *eif4e-bp1* expression (*p* > 0.05, Figure 4D).

### 3.7. Plasma Antioxidant Indices Under Heat Stress

**Figure 5:**
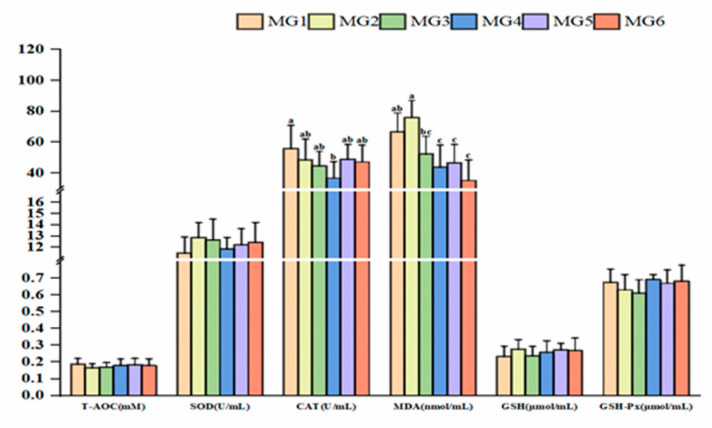
Expression of plasma antioxidant indices (n = 9). Statistically significant variations (*p* < 0.05) were indicated by distinct lowercase alphabetical superscripts, with unmarked values demonstrating no significant difference.

Figure 5 presents the results of plasma antioxidant indices. CAT activity peaked in the MG4 group, showing an initial decrease and a subsequent increasing trend (*p* < 0.05). Conversely, MDA levels showed a significant reduction in the MG4–MG6 groups (*p* < 0.05). However, no significant differences were observed in SOD, T-AOC, GSH, and GSH-Px indices (*p* > 0.05).

### 3.8. Genes Related to Immunity in the Gill Under Heat Stress

**Figure 6:**
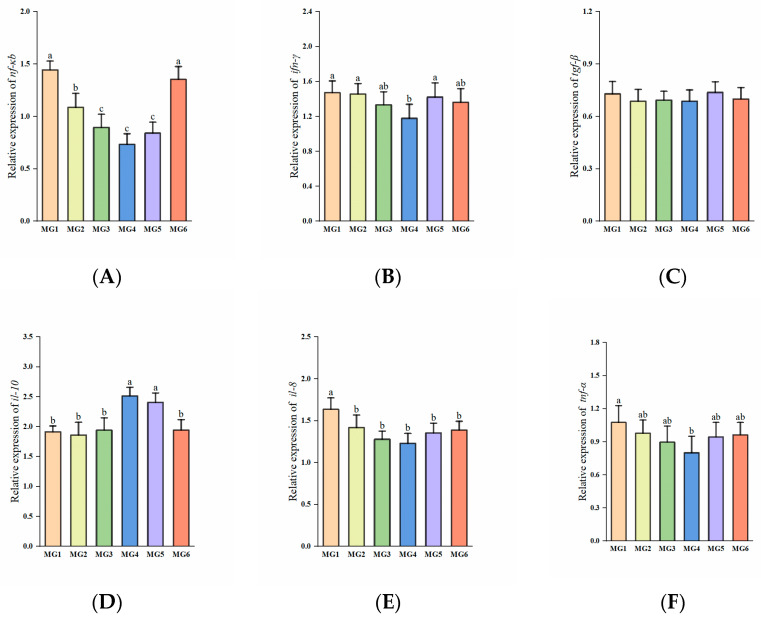
Expression of immune-related genes in the gill (n = 9): (**A**) *nf-κb*, (**B**) *ifn-γ*, (**C**) *igf-β*, (**D**) *il-10*, (**E**) *il-8*, (**F**) *tnf-α*. Statistically significant variations (*p* < 0.05) were indicated by distinct lowercase alphabetical superscripts, with unmarked values demonstrating no significant difference.

The expressions of *nf-κb*, *il-8*, and *tnf-α* showed a decreasing and then increasing trend, reaching the minimum in the MG4 group (*p* < 0.05, Figure 6A,E,F). The expression of *ifn-γ* in the MG4 group was significantly downregulated compared to the MG1, MG2, and MG5 groups (*p* < 0.05, Figure 6B). The expressions of *il-10* in the MG4 and MG5 groups showed marked elevation relative to other groups (*p* < 0.05, Figure 6D). No significant difference observed in *tgf-β* expression (*p* > 0.05, Figure 6C).

### 3.9. Plasma Ion Concentrations and Gill Na^+^/K^+^-ATPase Activity Under Heat Stress

Table 7 presents the results of plasma ion concentrations and gill Na^+^/K^+^-ATPase activity. The concentration of Na^+^ showed an initial increasing and subsequent decreasing trend (*p* > 0.05). Similarly, Cl^−^ concentrations demonstrated a comparable pattern of variation; however, a significant increase was observed in the MG4 group (*p* < 0.05). K^+^ concentration showed the opposite pattern, with significantly lower values in the MG4 group (*p* < 0.05). The concentration of Ca^2+^ in the MG1 group was markedly reduced relative to other groups (*p* < 0.05). The MG4 group exhibited peak Na^+^/K^+^-ATPase activity, displaying an initial increase followed by a subsequent decrease (*p* < 0.05).

### 3.10. Genes Related to Apoptosis and TUNEL Immunofluorescence Labeling Analysis in the Gill Under Heat Stress

**Figure 7:**
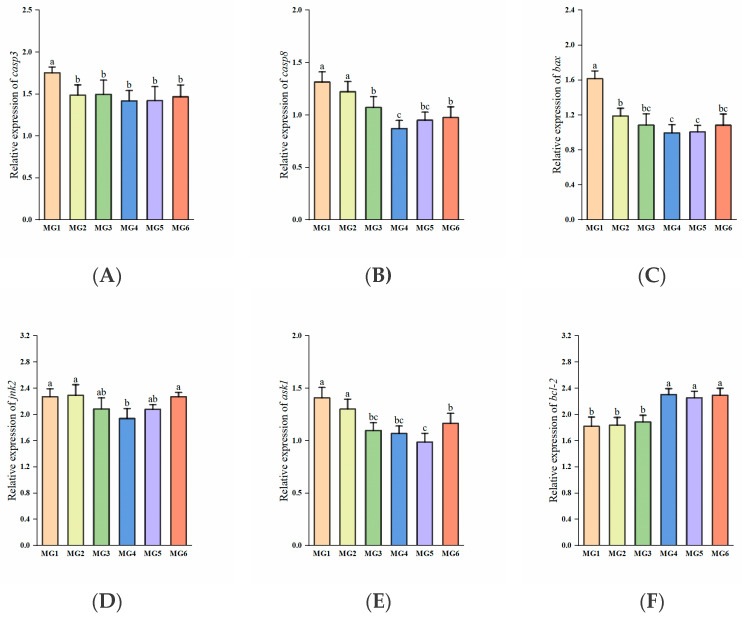
Expression of apoptosis-related genes in the gill (n = 9): (**A**) *casp3*, (**B**) *casp8*, (**C**) *bax*, (**D**) *jnk2*, (**E**) *ask1*, (**F**) *bcl-2*. Statistically significant variations (*p* < 0.05) were indicated by distinct lowercase alphabetical superscripts.

The expressions of *casp3* and bax exhibited significant downregulation in the MG2–MG6 groups, reaching minimal levels in the MG4 group (*p* < 0.05, Figure 7A,C). The MG4 group demonstrated the lowest *casp8* and jnk2 expressions, with the MG5 group showing the lowest *ask1* expression (*p* < 0.05, Figure 7B,D,E). In contrast, *bcl-2* expression displayed marked upregulation in the MG4-MG6 groups relative to the MG1–MG3 groups (*p* < 0.05, Figure 7F).

**Figure 8:**
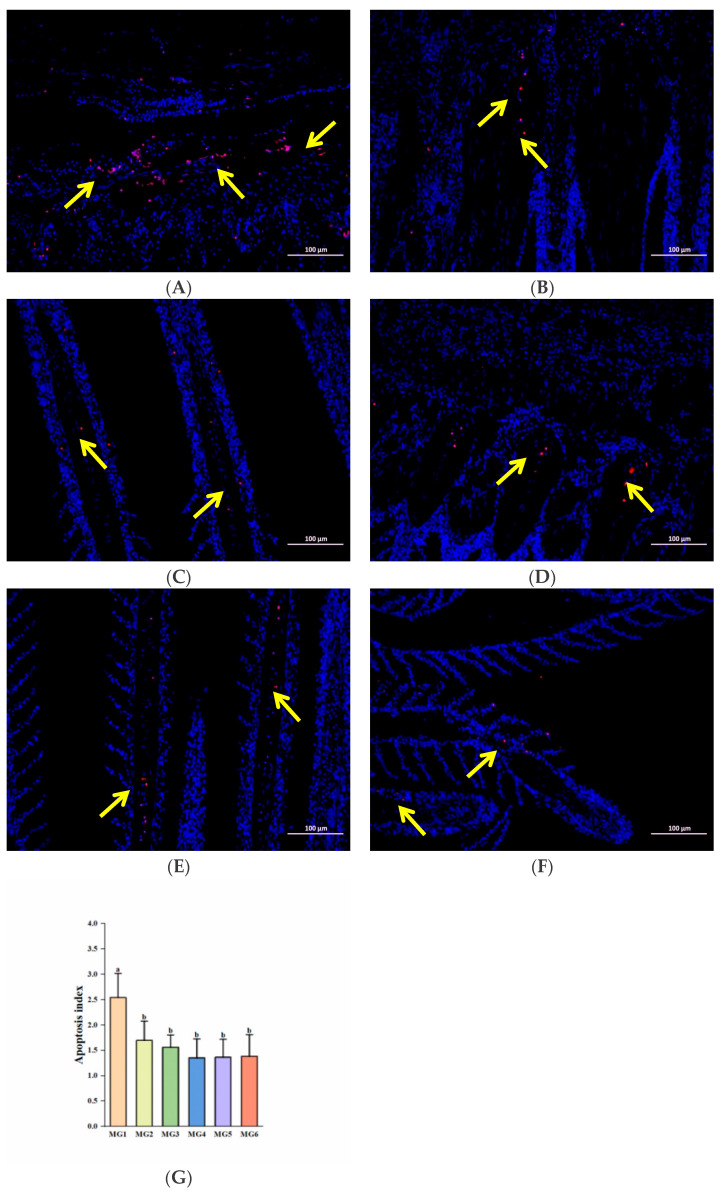
TUNEL immunofluorescence labeling analysis (n = 9): (**A**) MG1 group, (**B**) MG2 group, (**C**) MG3 group, (**D**) MG4 group, (**E**) MG5 group, (**F**) MG6 group, (**G**) Quantitative analysis of apoptosis index. The yellow arrow points to positive cells; the scale Bar is 100 μm. Statistically significant variations (*p* < 0.05) were indicated by distinct lowercase alphabetical superscripts.

Correspondingly, we observed that the number of positive cells (red color) was higher in the MG1 group, while the rest of the groups showed a significant decrease (*p* < 0.05, Figure 8A–F). And apoptotic indices demonstrated significant reductions in the MG2–MG6 groups (*p* < 0.05, Figure 8G).

## 4. Discussion

### 4.1. Effect of Mg on Growth Performance and Optimal Mg Requirement

Studies in freshwater fish have shown that a moderate increase in the supply of magnesium in the feed can promote fish growth, and that magnesium deficiency leads to growth inhibition [34,35]. This was confirmed by our findings that as the content of Mg in the feed increased from 1.01 g/kg to 2.24 g/kg, FBW, WGR, and SGR showed an upward trend and FCR showed a downward trend, showing a good growth promotion effect. Interestingly, we also found a decrease in growth performance when Mg content reached 2.35 g/kg and further decreased when it reached 2.51 g/kg. This led us to conclude that excessive Mg contents in feeds may adversely affect the growth of juvenile *M. salmoides*. This may be since high Mg increases the excretion burden of Mg in fish and can lead to impaired physiological functions [36,37]. This was confirmed by M. Musharraf’s study on rohu (*Labeo rohita*) [38] and Zhang’s study on *M. amblycephala* [2]. In addition, we obtained an optimal Mg requirement of 2.04 and 2.15 g/kg for juvenile *M. salmoides* by quadratic regression analysis of SGR and FCR. The Mg requirement of most farmed fish ranges from 0.4 to 0.6 g/kg [39], and our results were higher than most. This may be attributed to the following reasons. On the one hand, due to the different species and growth stages of fish. *M. salmoides* may utilize nutrients with different efficiency than other fish due to its fast growth rate and high metabolic demand [40,41]. The fish used in this study were 2.27 ± 0.02 g juveniles, which were in the rapid growth stage and had higher nutritional requirements [42]. On the other hand, there are differences in feed ingredients. MgSO_4_·7H_2_O used for Mg supplementation in this study is inorganic Mg, compared to organic Mg, which has higher solubility and bioavailability [43]. In addition, Mg in plant-based raw materials is mostly in the form of phytates, and Mg in fish meal is mostly in the form of phosphates and carbonates, which are also low utilized by fish [44]. This is similar to the results of a study in common carp (*Cyprinus carpio*), which found a significant increase in growth rate at dietary Mg contents up to 3.2 g/kg [45]. Therefore, a dietary Mg content of 2.24 g/kg is most favorable for the growth of juvenile *M. salmoides*.

### 4.2. Effect of Mg on Protein Metabolism

In our study of genes associated with protein metabolism in the liver, the expressions of *igf-1* were found to be significantly elevated in the MG4–MG6 groups, and *igf-1* has a promotional effect on protein synthesis [46]. Also, *igf*-1 is a key gene for growth promotion. Interestingly, it did not appear to activate the downstream *mtor* and *rps6k* genes. This also explained the lack of significant differences in fish whole-body protein content. Dietary Mg was also found not to promote protein synthesis in marine shrimp (*Litopenaeus vannamei*) [47] and soft-shell turtle (*Pelodiscus sinensis*) [48]. In summary, Mg supplementation did not significantly affect protein synthesis in this study. Nevertheless, significant differences in growth performance were found in our study. Factors such as improved energy availability, characterization of growth and developmental stages, reduced maintenance requirements, and changes in protein turnover may have contributed to this phenomenon and are not limited to protein deposition alone.

### 4.3. Effect of Mg on Glucose Metabolism

Mg is a key factor in controlling glucose. In our analysis of hepatic glucose metabolism-related gene expression, we found that *glut2* expressions were significantly elevated in the MG3–MG6 groups. Its elevated expression promotes insulin secretion [49], and inhibits gluconeogenesis through the PI3K/AKT signaling pathway [50]. The expressions of *g6pdh* were significantly higher in the MG4 and MG5 groups. *G6pdh* indirectly supports glycolysis through the metabolites of the phosphopentose pathway [51]. Despite the absence of significant changes, the expressions of *gk* and *pk*, which are closely related to glycolysis, were the highest in the MG4 group. *Pepck* and *g6pase*, as the key rate-limiting enzymes of gluconeogenesis [52], the expressions of *pepck* showed a tendency to decrease after Mg supplementation, and the expression of *g6pase* was significantly lower in the MG5 group than in the MG1 group. Similar phenomena were found in studies on *M. amblycephala* [2]. In addition, plasma biochemical indices suggested that dietary Mg supplementation has a positive effect on reducing plasma GLU. Studies have shown that Mg deficiency may lead to a blockage in the process of glucose metabolism, making plasma glucose levels relatively high [53]. Moderate amounts of Mg can enhance insulin secretion and sensitivity, and promote glucose uptake and utilization, thus lowering plasma GLU levels [54]. In short, Mg addition may promote glycolysis, inhibit gluconeogenesis, improve glucose metabolism, and result in lower blood glucose, and was best in the MG4 and MG5 groups.

### 4.4. Effect of Mg on Lipid Metabolism

In our study of genes related to hepatic lipid metabolism, we found that the expressions of *ppar-α* were significantly decreased in the MG3-MG6 groups, and there was no significant change in *cpt1* (downstream of *ppar-α*) expressions. PPAR-α plays a key role in fatty acid oxidation and promotes lipolysis [55], which suggests that lipolysis is inhibited. In contrast, the expressions of *ppar-γ*, *fas,* and *elovl2* were significantly increased in the MG3–MG6 groups, and the expressions of *acc* were also significantly increased in the MG4–MG6 groups. Up-regulation of the expressions of *ppar-γ*, *fas*, *elovl2,* and *acc* can promote lipid synthesis and storage [56]. This also explained that the fish whole-body crude lipid content was significantly higher in the MG6 group. This phenomenon of whole-body lipid deposition with increasing Mg content in the diet was also found in studies on *H. fossilis* [11] and guppy (*Poecilia reticulata* Peters) [57]. However, in terms of plasma lipid, we found that Mg supplementation lowered plasma TG levels, which contradicts the phenomenon of lipid deposition in fish whole-body. This may be because insulin resistance is closely related to hypertriglyceridemia [58], and Mg, by improving insulin sensitivity, may have indirectly reduced plasma TG levels. Moreover, certain long-chain unsaturated fatty acids are more readily absorbed and stored by tissues rather than entering the blood circulation [59]. In this case, plasma TG levels may be reduced due to the type and distribution of fatty acids. Similarly, the results of Tongyai [60] and Chaudhary [61] on rats showed that Mg deficiency leads to elevated plasma levels of TG. In addition, Mg supplementation resulted in a decrease in plasma LDL-C, an increase in plasma HDL-C, and no significant change in plasma TC, indicating that Mg supplementation improved lipid health in *M. salmoides*. Excessive LDL-C leads to cholesterol deposition in blood vessel walls, whereas HDL-C helps to remove cholesterol from blood vessels. The reason for the lack of significant changes in plasma TC levels, we guess, is because the decrease in plasma LDL-C and the increase in plasma HDL-C may cancel each other out. Therefore, feed supplementation with Mg may increase lipid levels in juvenile *M. salmoides* (highest in the MG6 group), but did not affect their plasma lipid health.

### 4.5. Effect of Mg on Plasma Antioxidant Capacity and Gill Immunity Under Heat Stress

Generally, elevated temperature leads to oxidative stress in fish and enhances ROS production [62]. CAT activity is usually increased due to the regulation of ROS and is a scavenger of ROS [63]. In our study, Plasma CAT activity was significantly lower in the MG4 group, which may be because the antioxidant system in the MG4 group was in an efficient and stable equilibrium, with a lower degree of oxidative stress and less ROS production. In addition, plasma MDA contents were significantly lower in the MG4–MG6 groups. MDA is a product of lipid peroxidation, and a decrease in its content indicates attenuation of lipid peroxidation damage to the cell membrane [64]. A similar phenomenon was observed in Zhang’s study [2]. These results indicated that the MG4 group exhibited the most pronounced improvement in antioxidant-related indices, which may reflect a potential enhancement of antioxidant capacity in juvenile *M. salmoides* under heat stress. Oxidative stress under high temperature can lead to immune imbalance in fish, and studies have shown that Mg has a favorable anti-inflammatory effect. In our study, the expressions of *nf-κb*, *il-8*, *tnf-α*, and *ifn-γ* decreased to different degrees relative to the MG1 group and were the lowest in the MG4 group. NF-κB is a core transcription factor of the inflammatory response, which is activated in heat stress, and directly drives the expression of downstream pro-inflammatory genes (*tnf-α*, *il-8*) and indirectly affects the expression of *ifn-γ* [65]. Our study demonstrated that moderate amounts of Mg inhibited *nf-κb* expression and did not promote inflammation. In addition, the expressions of *il-10* (the key anti-inflammatory gene) were up-regulated in the MG4 and MG5 groups, which corroborated the anti-inflammatory effect of Mg. Although we did not find studies on the effect of Mg on immune-related genes in fish, the results were similar to those of related studies in mammals [66]. Overall, the MG4 group showed the best immunocompetence.

### 4.6. Effect of Mg on Plasma Ion Concentrations and Gill Na^+^/K^+^-ATPase Activity Under Heat Stress

Heat stress significantly affects ionic homeostasis and the activity of Na^+^/K^+^-ATPase, a key enzyme for maintaining ionic homeostasis and osmolarity stability in fish [28,67]. In this experiment, the Na^+^/K^+^-ATPase activity was significantly increased and then significantly decreased, reaching the highest in the MG4 group. In addition, the MG4 group had significantly higher concentrations of Na^+^ and Cl^−^, and significantly lower concentrations of K^+^. This may be due to the fact that elevated Na^+^/K^+^-ATPase activity accelerates the transport of Na^+^ from intracellular to extracellular, which may lead to higher plasma concentrations of Na^+^, the opposite is true for K^+^ [68]. Cl^−^ concentration is generally positively correlated with sodium ion concentration. The results of Huang’s study on tilapia (GIFT; *Oreochromis niloticus*) in low-temperature stress [29] and Nathan’s study on *M. salmoides* in a high-iron environment [69] were similar to ours. And studies have shown that Mg can affect the absorption and metabolism of Ca [70]. Dietary Mg supplementation in our study significantly increased plasma Ca^2+^ concentration and improved Ca and Mg balance. It suggests that moderate supplementation of dietary Mg can effectively increase the vitality of the Na^+^/K^+^-ATPase and improve ionic homeostasis.

### 4.7. Effect of Mg on Gill Apoptosis Under Heat Stress

Heat stress also induces apoptosis [71], a programmed cell death under conditions such as embryonic development and pathology [72]. In addition to oxidative stress and Na^+^/K^+^-ATPase activity affecting apoptosis, it is also closely regulated by apoptosis-related genes. The *casp8* activates *casp3* through an exogenous pathway, and *casp3* is the final executioner of apoptosis [73]. In our study, the expressions of both *casp3* and *casp8* were reduced in the MG2–MG6 groups and lowest in the MG4 group. In addition, the expression of *jnk2* in the MG4 group and *ask1* in the MG5 group was also the lowest. And *ask1* can activate *jnk2*, and jointly participate in stress-induced apoptosis [74]. Similarly, the pro-apoptotic *bax* expressions were decreased in the MG2–MG6 groups and were lowest in the MG4 group. The *bcl-2* is an anti-apoptotic member of the BCL2 family [75], and *bcl-2* expressions were significantly elevated in the MG4–MG6 groups. We also found that Mg deficiency in feed aggravated apoptosis [30], which is similar to our findings. In addition, we also found that Mg supplementation significantly reduced the apoptotic index in gills by TUNEL immunofluorescence labeling analysis, which is consistent with the results of gene analysis. The degree of apoptosis also reflects, to some extent, the tolerance of fish to adverse environments [76]. Based on the combined experimental results, this study preliminarily suggests that Mg supplementation in feed may exert a certain inhibitory effect on the apoptosis process in juvenile *M. salmoides* under heat stress, and the MG4 group performed the best.

### 4.8. Limitations and Prospects

First is the breadth of the research, namely the development of *M. salmoides*. This study primarily focuses on the juvenile stage of *M. salmoides*. Since nutritional requirements may vary across different growth stages of the same fish species, future research should encompass all growth phases of *M. salmoides* cultivation to obtain more precise and targeted experimental data. Second is the depth of the research, namely, the further exploration of the underlying mechanisms. This study primarily focused on testing changes in enzyme activity and gene expression related to Mg. It has not yet comprehensively and clearly addressed other physiological function indicators in *M. salmoides* nor elucidated specific mechanisms of action. Therefore, subsequent research should also employ omics approaches to screen differentially expressed genes, clarify the relationships between signaling pathways affecting different indicators, and further elucidate the physiological mechanisms by which Mg exerts its effects at the molecular level. Finally, the Mg source used in this study was MgSO_4_·7H_2_O, an inorganic Mg compound. It may be worthwhile to explore the use of organic Mg or nano-Mg as alternative sources to investigate whether they yield superior growth and developmental outcomes for juvenile *M. salmoides*.

## 5. Conclusions

The results of the current study indicate that appropriate Mg levels (2.24 g/kg) in feeds could be effective in promoting growth and improving glucose metabolism, but lead to lipid deposition. Based on the quadratic regression analysis of SGR and FCR, the optimal Mg requirement for juvenile *M. salmoides* was 2.04 and 2.15 g/kg, respectively. In addition, the Mg level of 2.24 g/kg under heat stress enhanced the antioxidant and immune capacity and improved ionic homeostasis while inhibiting apoptosis in juvenile *M. salmoides*. These findings provide important guidance for metabolic regulation in carnivorous fish and underscore the significance of Mg supplementation in feed for high-temperature aquaculture.

## Figures and Tables

**Table 1:** Experimental basic formula (% dry matter).

Ingredients	Level (%)	Ingredients	Level (%)
Fish meal ^1^	15.00	Choline chloride	0.50
Casein ^2^	31.20	Vitamin premix ^4^	1.00
Gelatine ^3^	7.80	Mineral premix ^5^	1.00
Wheat flour ^1^	16.00	Monocalcium phosphate	4.00
Fish oil ^1^	4.50	Microcrystalline cellulose	14.40
Soybean oil ^1^	4.50	Vitamin C	0.10
Analyzed proximate composition (dry matter)			
Crude protein (%)	47.51 ± 0.70		
Crude lipid (%)	10.55 ± 0.19		
Gross energy (KJ/g)	15.38 ± 0.09		

^1^ The crude protein contents of fish meal and wheat flour were 67.8% and 13.1%, respectively. The crude lipid contents of fish meal, wheat flour, fish oil, and soybean oil were 9.3%, 4.0%, 100.0%, and 100.0%, respectively. Biomar Tongwei Biotech Co., Ltd. (Wuxi, China) provided them. ^2^ Merck Drugs & Biotechnology Co., Ltd. (Darmstadt, Germany) provided casein (crude protein 90.0%). ^3^ Shanghai Zhanyun Chemical Co., Ltd. (Shanghai, China) provided gelatin (crude protein 90.3%). ^4^ Hanove Biotechnology Co., Ltd. (Wuxi, China) provided Vitamin premix. ^5^ Mineral premix (no magnesium) was self-configured.

**Table 2:** Chemical analysis items.

Items	Methods	Assay Kits	Testing Equipment
Total protein (TP)	International Federation of Clinical Chemistry recommended	Mindray Medical International Ltd. (Shenzhen, China).	Mindray BS-400 automatic biochemical analyzer (Mindray Medical International Ltd., Shenzhen, China).
Glucose (GLU)			
Total cholesterol (TC)			
Triglyceride (TG)			
Low-density lipoprotein cholesterol (LDL-C)			
High-density lipoprotein cholesterol (HDL-C)			
Superoxide dismutase (SOD)	WST-1 method	Jian Cheng Bioengineering Institute (Nanjing, China)	Spectrophotometer (Thermo Fisher Multiskan GO, Shanghai, China).
Glutathione (GSH)	Microplate method		
Total antioxidant capacity (T-AOC)	ABTS method		
Malondialdehyde (MDA)	TBA method		
Catalase (CAT)	Ammonium molybdenum acid method		
Glutathione peroxidase (GSH-Px)	Colorimetric method		
Na^+^	Colorimetric method		
Na^+^/K^+^-ATPase	Colorimetric method		
K^+^	Microplate method		
Cl^-^	Microplate method		
Ca^2+^	Microplate method		
Moisture	Dry at 105 °C under atmospheric pressure until constant weight is achieved.	-	Electric blast drying oven (Shanghai Yiheng Scientific Instrument Co., Ltd., Shanghai, China)
Ash	Carbonize, then incinerate at 560 °C for 5 h.		XL-2A muffle furnace (Hangzhou, China)
Real-time polymerase chain reaction (RT-PCR)	-	One-Step SYBR^®^ PrimeScript™ PLUS RT-PCR Kit (Takara, Dalian, China)	CFX96 Real-Time PCR Detection System (Bio-Rad Laboratories, Shanghai, China)

**Table 3:** Primer sequences.

Genes	Forward Primer (5′–3′)	Reverse Primer (5′–3′)	Source
Glyceraldehyde-3-phosphate dehydrogenase (*gapdh*)	ACTGTCACTCCTCCATCTT	CACGGTTGCTGTATCCAA	AZA04761.1
Pyruvate kinase (*pk*)	CACGCAACACTGGCATCATC	TCGAAGCTCTCACATGCCTC	MT431526.1
Glycerate kinase (*gk*)	CCCTTGTGGGCAGGAGAAAA	ACAACTGAGTCCTCCTTGCG	XP_023260296.1
Phosphoenolpyruvate carboxykinase (*pepck*)	GGCAAAACCTGGAAGCAAGG	ATAATGGCGTCGATGGGGAC	MT431525.1
Glucose 6-phosphatase (*g6pase*)	ACACAGCAGCCCTGTTCTAC	CCGTTCACACAGTACGGGAT	XM_038735542.1
Glucose transporter 2 (*glut2*)	TCACCGTGTTTATTTATCTTCG	AGCTCCGTATCGTCTTTGG	XM_038728860
Glucose 6-phosphate dehydrogenase (*g6pdh*)	AGTCCAGTCACTCCAACA	TCTCTGAATAACCACCACAA	CAG04059.1
Elongation of very long Chain fatty acids protein 2 (*elovl2*)	GGACACAACAATACAAGATGG	GAACAGGTAGCACAGCAAT	Cluster-21914.20999
Peroxisome proliferator-activated receptor-α *(ppar-α*)	AGGCTTCATCACCAGAGA	TCCGCAGCAGATAATAGTAG	MK614719.1
Peroxisome proliferator-activated receptor-γ (*ppar-γ*)	GAGTTCTCAGTCAAGTTCAAC	AATGTAGCACCGTCTCCT	MK614721.1
Acetyl-CoA carboxylase *(acc*)	TTACATCGCAGCCAACAG	CTCTCCACCTTCCTCTACA	XP_022609673.1
Fatty acid synthase (*fas*)	AGTTGAAGGCTGCTGATG	GCTGTGGATGATGTTGGT	XP_028423094.1
Carnitine O-palmitoyltransferase 1 (*cpt1*)	TTACCGTATGGCTATGACTG	GGCTCCGATAACACCTCT	XP_027141042.1
Mechanistic target of rapamycin (*mtor*)	TTTGGAACCAAACCCCGTCA	ATCAGCTCACGGCAGTATCG	XM_038723321.1
Ribosomal protein S6 (*rps6*)	TCCAGAGACTCGTGACACCT	AGCTTGGCATACTCTGAGGC	XM_038713349.1
Eukaryotic translation initiation factor 4E-binding protein 1 (*eif4e-bp1*)	CCAGGATCATCTATGACCGAAAG	TGCAGCGATATTGTTGTTGTTC	XM_038703879.1
Insulin-like growth factor-1 (*igf-1*)	CCTCTGCCTGTGTATAATCA	TGTCCGTCTTAGCCATCT	XM_038738328.1
Nuclear factor-kappa B (*nf-κb)*	CCACTCAGGTGTTGGAGCTT	TCCAGAGCACGACACACTTC	XP_027136364.1
Interleukin-10 (*il-10)*	CGGCACAGAAATCCCAGAGC	CAGCAGGCTCACAAAATAAACATCT	XM_038696252.1
Transforming growth factor-β (*tgf-β*)	CACCAAGGAGATGCTGATT	CGTATGTTAGAGATGCTGAAG	XM_038693206.1
Interferon-γ (*ifn-γ*)	GAGCAAAGCATTGTGGGAGC	AGATGAGTTTTGGCCCTCCG	XM_038707474.1
Caspase-3 (*casp3*)	GAGGCGATGGACAAGAGTCA	CACAGACGAATGAAGCGTGG	XM_038713063.1
Caspase-8 (*casp8*)	GAGACAGACAGCAGACAACCA	TTCCATTTCAGCAAACACATC	XM_038726463.1
Bcl-2 associated X protein (*bax*)	ACTTTGGATTACCTGCGGGA	TGCCAGAAATCAGGAGCAGA	XM_038704178.1
B-cell lymphoma-2 (*bcl-2*)	TGTGGGGCTACTTTTTGGCA	TTCGACTGCCACCCCAATAC	PRJNA725023 [33]
Apoptosis signal regulating kinase 1 (*ask1*)	CAACTACGCCTTCATCCCGT	GGTCCCAACAGCATCTCGAA	PRJNA725023 [33]
C-Jun N-terminal kinase 2 (*jnk2*)	GTCTTCTCCCTTCACCGCTC	CGTGACAGCCGGTTTTCCTA	PRJNA725023 [33]
Interleukin-8 (*il-8*)	GAGGGTACATGTCTGGGGGA	CCTTGAAGGTTTGTTCTTCATCGT	XM_038713529.1
Tumor necrosis factor-α (*tnf-α*)	CTTCGTCTACAGCCAGGCATCG	TTTGGCACACCGACCTCACC	XM_038710731.1

**Table 4:** Growth performance of juvenile *M. salmoides*.

Diets	IBW (g)	FBW (g)	WGR (%)	SGR (%/d)	FCR
MG1	2.27 ± 0.02	15.27 ± 0.56 ^c^	569.04 ± 31.81 ^b^	3.02 ± 0.08 ^b^	1.50 ± 0.05 ^a^
MG2	2.28 ± 0.01	15.55 ± 0.27 ^bc^	582.85 ± 9.17 ^b^	3.05 ± 0.02 ^b^	1.48 ± 0.03 ^ab^
MG3	2.28 ± 0.02	16.60 ± 0.70 ^abc^	629.45 ± 36.81 ^ab^	3.15 ± 0.08 ^ab^	1.41 ± 0.03 ^bc^
MG4	2.27 ± 0.01	17.41 ± 0.26 ^a^	667.59 ± 14.20 ^a^	3.23 ± 0.03 ^a^	1.37 ± 0.03 ^c^
MG5	2.26 ± 0.01	16.72 ± 0.23 ^ab^	640.33 ± 11.46 ^ab^	3.18 ± 0.02 ^ab^	1.41 ± 0.02 ^abc^
MG6	2.27 ± 0.03	16.05 ± 0.53 ^bc^	608.78 ± 28.26 ^ab^	3.11 ± 0.06 ^ab^	1.41 ± 0.01 ^bc^

Data were presented as mean ± SD (n = 3). Statistically significant variations (*p* < 0.05) were indicated by distinct lowercase alphabetical superscripts, with unmarked values demonstrating no significant difference. WGR (%) = 100 × (FBW (g) − IBW (g))/IBW (g). SGR (%/d) = 100 × [(Ln (FBW (g)) − Ln (IBW (g)))/days]. FCR = dry feed fed (g)/(FBW (g) − IBW (g)).

**Table 5:** Whole-body composition of juvenile *M. salmoides*.

Diets	Moisture (%)	Crude Protein (%)	Crude Lipid (%)	Ash (%)
MG1	70.97 ± 0.23	16.17 ± 0.08	9.23 ± 0.08 ^b^	3.43 ± 0.11
MG2	70.79 ± 1.29	16.20 ± 0.06	9.72 ± 0.15 ^ab^	3.28 ± 0.06
MG3	69.69 ± 0.80	16.16 ± 0.14	9.92 ± 0.60 ^ab^	3.23 ± 0.15
MG4	70.09 ± 0.70	16.36 ± 0.23	10.12 ± 0.54 ^ab^	3.30 ± 0.13
MG5	69.77 ± 0.55	16.13 ± 0.15	10.28 ± 0.33 ^ab^	3.21 ± 0.11
MG6	69.57 ± 1.71	16.36 ± 0.03	10.32 ± 0.35 ^a^	3.19 ± 0.12

Data were presented as mean ± SD (n = 9). Statistically significant variations (*p* < 0.05) were indicated by distinct lowercase alphabetical superscripts, with unmarked values demonstrating no significant difference.

**Table 6:** Plasma biochemical indices of juvenile *M. salmoides*.

Diets	GLU (mmol/L)	LDL-C (mmol/L)	HDL-C (mmol/L)	TP (g/L)	TG (mmol/L)	TC (mmol/L)
MG1	16.14 ± 1.94 ^a^	2.63 ± 0.45 ^a^	2.23 ± 0.28 ^b^	26.14 ± 2.82	11.76 ± 1.75 ^a^	4.63 ± 0.78
MG2	14.62 ± 2.93 ^ab^	1.72 ± 0.42 ^b^	3.37 ± 0.59 ^a^	28.48 ± 5.37	5.17 ± 1.49 ^b^	4.45 ± 0.66
MG3	15.72 ± 2.11 ^a^	1.97 ± 0.25 ^b^	3.15 ± 0.61 ^a^	27.55 ± 3.58	5.78 ± 0.84 ^b^	4.78 ± 0.91
MG4	12.11 ± 1.17 ^b^	1.75 ± 0.44 ^b^	3.29 ± 0.45 ^a^	30.70 ± 2.12	5.31 ± 0.82 ^b^	5.13 ± 1.07
MG5	13.44 ± 1.46 ^ab^	1.81 ± 0.43 ^b^	3.41 ± 0.46 ^a^	30.13 ± 3.83	4.98 ± 1.44 ^b^	4.40 ± 0.47
MG6	14.89 ± 3.09 ^ab^	2.00 ± 0.31 ^b^	3.28 ± 0.59 ^a^	29.25 ± 3.21	5.37 ± 1.22 ^b^	4.53 ± 0.44

Data were presented as mean ± SD (n = 9). Statistically significant variations (*p* < 0.05) were indicated by distinct lowercase alphabetical superscripts, with unmarked values demonstrating no significant difference.

**Table 7:** Plasma ion concentrations and gill Na^+^/K^+^-ATPase activity of juvenile *M. salmoides*.

Diets	Na^+^ (mmol/L)	K^+^ (mmol/L)	Cl^−^ (mmol/L)	Ca^2+^ (mmol/L)	Na^+^/K^+^-ATPase (U/mgprot)
MG1	134.63 ± 10.29	2.94 ± 0.40 ^a^	144.33 ± 10.26 ^b^	0.69 ± 0.08 ^b^	0.22 ± 0.04 ^d^
MG2	137.90 ± 9.28	2.93 ± 0.22 ^ab^	157.55 ± 15.78 ^ab^	1.07 ± 0.16 ^a^	0.48 ± 0.08 ^c^
MG3	147.39 ± 8.27	2.54 ± 0.42 ^ab^	157.41 ± 7.64 ^ab^	1.00 ± 0.18 ^a^	0.73 ± 0.15 ^b^
MG4	147.35 ± 6.90	2.43 ± 0.47 ^b^	161.92 ± 7.37 ^a^	1.10 ± 0.21 ^a^	1.81 ± 0.15 ^a^
MG5	143.21 ± 11.02	2.53 ± 0.18 ^ab^	158.49 ± 10.56 ^ab^	1.08 ± 0.26 ^a^	0.75 ± 0.10 ^b^
MG6	133.37 ± 11.68	2.63 ± 0.28 ^ab^	147.54 ± 14.54 ^ab^	1.02 ± 0.15 ^a^	0.16 ± 0.04 ^d^

Data were presented as mean ± SD (n = 9). Statistically significant variations (*p* < 0.05) were indicated by distinct lowercase alphabetical superscripts, with unmarked values demonstrating no significant difference.

## Data Availability

Data will be made available on request.
